# Effectiveness of Bariatric Surgery Versus Nutritional Interventions in Adolescents: A Retrospective Cohort Study

**DOI:** 10.1007/s11695-026-08521-8

**Published:** 2026-02-28

**Authors:** Miri Mizrahi Reuveni, Bar Cohen, Dor Atias, Ilan Yehoshua, Shelley A Sternberg, Eduardo Schejter, Zorian Radomyslsky, Yakov Segal, Limor Tal Pony, Galit Kowen Sandbank, Joseph Azuri, Limor Adler

**Affiliations:** 1 Dina Recanati School of Medicine, Reichman University, Herzliya, Israel; 2 Health Division, Maccabi Health Care Services, Tel Aviv, Israel; 3 Gray❜s Faculty of Medical & Health Sciences, Tel Aviv University, Tel Aviv, Israel; 4 Faculty of Medicine, Ariel University, Ariel, Israel; 5 Gray❜s Faculty of Medical & Health Sciences, Tel Aviv University, Tel Aviv, Israel; 6 Health Division, Maccabi Health Care Services, Tel Aviv, Israel

**Keywords:** Bariatric surgery, Adolescents, Obesity, Primary care, Vitamin deficiencies

## Abstract

**Introduction:**

Bariatric surgery offers sustained weight loss for adolescents with severe obesity, but long-term data on its effectiveness is limited. Our objective was to compare outcomes in adolescents who underwent bariatric surgery versus those who received nutritional intervention.

**Methods:**

We conducted a retrospective cohort study of individuals under 18 who underwent bariatric surgery within < institution name> between 2011 and 2021. A comparison group included adolescents with class II obesity or higher and who had ≥ 3 dietitian visits. Anthropometric measures and blood test results (hemoglobin, TSH, vitamin D, folic acid, B12) were collected over five years. Multivariate linear mixed models assessed group differences over time.

**Results:**

The cohort included 278 adolescents: 152 in the surgery group and 126 in the nutritional group. Over five years, BMI decreased from 44 to 32 in the surgery group but remained largely unchanged in the nutritional group (45.5 to 44), *p* < 0.001. At year 5, the surgery group had lower hemoglobin (12.45 vs. 13.46, *p* < 0.001) and declining B12 levels (*p* < 0.001). TSH levels decreased modestly in both groups, more so in the surgery group (*p* = 0.04).

**Conclusions:**

Bariatric surgery in adolescents led to significant, sustained BMI reductions but was associated with declines in some nutritional markers, highlighting the need for ongoing monitoring.

**Supplementary Information:**

The online version contains supplementary material available at 10.1007/s11695-026-08521-8.

## Key Points

• Bariatric surgery in adolescents with severe obesity proved effective in reducing weight and BMI, compared to nutritional intervention.

• In the five-year period after bariatric surgery, adolescents had markedly lower levels of hemoglobin, vitamin B12, and TSH.

• Micronutrient deficiencies are a potential risk of bariatric surgeries, requiring long-term monitoring and supplementation.

**Supplementary Information:**

The online version contains supplementary material available at 10.1007/s11695-026-08521-8.

## Introduction

The prevalence of severe obesity among adolescents has increased substantially over recent decades. Current estimates indicate that approximately 9.5% of U.S. adolescents and 2–5% of adolescents worldwide meet criteria for severe obesity (class II obesity, BMI ≥ 120% of the 95th percentile) [1, 2]. This trend is associated with significant physical and psychological comorbidities, including type 2 diabetes, hypertension, dyslipidemia, and depression [3, 4]. Adolescents with obesity are also at increased risk for low self-esteem, body image dissatisfaction, and isolation [5]. These psychological burdens often contribute to anxiety, depression, and disordered eating behaviors, which can persist into adulthood and complicate treatment efforts [6].

The first line in obesity management is lifestyle changes, such as diet and exercise [7]. Additional therapeutic options include medication and surgery. However, conventional interventions such as lifestyle modification, behavioral therapy, and pharmacotherapy often result in modest and unsustainable weight loss among adolescents with severe obesity [8, 9]. Notably, newer-generation anti-obesity medications (e.g., GLP-1 receptor agonists and related agents) have demonstrated markedly improved weight-loss outcomes in adolescents compared with older pharmacotherapies, and these benefits appear sustainable with continued use [10, 11].

Although initially used for adults, growing evidence supports the safety and efficacy of bariatric procedures in adolescents, with studies demonstrating lasting weight loss, resolution of comorbidities, and improved quality of life [12, 13]. Given its irreversible nature and potential complications, bariatric surgery is approached with caution in adolescents [14]. Consequently, it is not widely performed in this group, with lower utilization rates compared to adults [15]. Guidelines for bariatric surgery before age 18 indicate this is an appropriate option for class II obesity with one significant obesity-related comorbidity or for class III obesity (≥ 140% of the 95th percentile) [16, 17]. Among the various bariatric procedures, laparoscopic sleeve gastrectomy (LSG) became the most performed in adolescents, due to its favorable risk profile, technical simplicity, and comparable outcomes to gastric bypass [18]. Laparoscopic sleeve gastrectomy involves removing approximately 70–80% of the stomach along the greater curvature, creating a narrow tubular gastric “sleeve.” The procedure reduces stomach capacity and decreases the number of ghrelin-producing cells, contributing to reduced appetite and early satiety [19, 20 ].

One risk of this operation is nutritional deficiencies resulting from reduced vitamin absorption. A five-year follow-up cohort study of 226 adolescents (ages 13–19) after Roux-en-Y Gastric Bypass (RYGB) or LSG found that those who had an RYGB had significantly lower B12 levels over time [21]. Ferritin and transferrin levels, however, decreased in both groups. Due to this risk, patients after bariatric surgery require supplementation; however, many struggle to adhere [22, 23]. Despite accumulating evidence, real-world data (i.e., routine clinical practice rather than controlled trial) on adolescent bariatric outcomes outside the U.S., particularly over extended periods, remain scarce.

In < country name>, bariatric surgeries are approved and subsidized for adolescents (13–18) in patients with class II obesity and serious obesity-related comorbidities, or patients with class III obesity and mild obesity-related comorbidities [24]. Patients must be over 13 years old and have a bone age of at least 15 in boys and 13 in girls, indicating completion of at least 95% of potential growth [24]. Post-operative management included routine nutritional supplementation for all patients following bariatric surgery. Nutritional intervention serves as the alternative therapeutic option. Adolescents can meet individually with a registered dietitian or participate in a structured group program, depending on personal preference. Participation and frequency of visits are determined by the adolescent and their parents, based on willingness and engagement. Each session lasts approximately 30 min. Both interventions are included in the national health basket and are largely state-subsidized, resulting in minimal out-of-pocket costs for patients. The aim of this study was to evaluate the long-term outcomes of bariatric surgery in adolescents over a five-year follow-up period, focusing on weight and laboratory parameters (hemoglobin, TSH, vitamin B12, vitamin D, and folic acid). Outcomes were compared with those of adolescents with severe obesity who underwent nutritional intervention.

## Materials & Methods

### Study Design & Setting

This is a retrospective cohort study. We collected data on all < institution name> members who underwent bariatric surgery before age 18, between 2011 and 2021. This group was compared to a group with similar baseline characteristics, including obesity class II or higher, who did not undergo bariatric surgery and received nutritional intervention. To ensure meaningful exposure to the nutritional intervention, only adolescents who attended at least three dietitian sessions were included in the study. Information regarding whether surgical intervention was offered to or declined by this cohort was not available. The total number of sessions varied according to the adolescents’ and parents’ engagement and adherence. This threshold does not reflect a clinical protocol.

We conducted a five-year follow-up to evaluate the long-term effects. For the nutritional intervention group, the index date was the first dietitian visit, and for the surgery group, the date of surgery. The study was approved by < institution name> Ethical Committee (IRB) and conducted in accordance with the principles of the Declaration of Helsinki (#0003-23-< institution name>, April 3rd, 2023).

### Participants

In the surgery group, we included all individuals who underwent bariatric surgery at < institution name > for the first time before age 18. All individuals had LSGs. In the nutritional intervention group, we included all individuals under 18 years of age with obesity class II or higher who received at least 3 dietitian visits.

### Variables

We collected sociodemographic data, including socioeconomic status (SES), gender, age, ethnic background, and residence in the periphery. We also collected medical information: initial weight, height, BMI, revision surgery, and comorbidities. We collected weight and BMI data throughout the study period, along with blood test results (hemoglobin, TSH, vitamin D, folic acid, and vitamin B12). Although data for other micronutrients were available in the medical records, we restricted the analysis to these specific markers as they represent the standard nutritional screening panel in routine primary care practice. These were summarized yearly. For years with multiple test results, the average value was calculated.

### Statistical Analysis

For descriptive statistics, we report numbers and percentages for categorical variables and means and standard deviations (SD) for continuous variables. To examine the association between surgery and health outcomes, we fitted multivariate linear mixed models, accommodating repeated measurements and enabling assessment of changes over time.

To explore the temporal trends, we included an interaction term between group allocation and year, allowing us to evaluate differences in changes over time between the groups. For the anthropometric metrics (BMI and weight), we present standard deviation scores (SDS), which are more suitable for children. Accordingly, we excluded measurements taken after age 19, resulting in the exclusion of 90 measurements. We believe it is warranted, as the accuracy and use of SDS improve the quality of the results. For the BMI and weight multivariate analyses, we performed two analyses due to lack of linearity (from index date to 2 years and from 2 to 5 years after index date). To best represent the trends, we incorporated the two models into a graphic display. Analyses performed using R v.4.4.2.

## Results

### Participants

The study included 278 patients: 152 underwent bariatric surgery and 126 received nutritional intervention. At the index date, the surgery group was older (16.25 vs. 15.15, *p* < 0.001) and mostly female (63%). Few patients in the surgery group resided in the periphery (29%, *n* = 44). Type 2 diabetes prevalence was similar between groups (2% vs. 0%, *p* = 0.7), but pre-diabetes was more prevalent among the surgery group (43% vs. 20%, *p* < 0.001). Baseline data are presented in Table 1.

**Table 1:** Baseline characteristics of adolescents who underwent bariatric surgery compared to those who received nutritional intervention only

Characteristic	Bariatric Surgery Group *N* = 152^1^	Nutritional Intervention Group *N* = 126^1^	*p*-value^2^
Age	16.25 (0.94)	15.17 (1.43)	< 0.001
Sex (M)	56 (37%)	59 (47%)	0.092
Socioeconomic status			
Low	44 (29%)	50 (40%)	0.2
Medium	71 (47%)	48 (38%)	
High	37 (24%)	28 (22%)	
Ethnic background			0.001
General	116 (79%)	76 (64%)	
Arab	5 (3.4%)	19 (16%)	
Ultra-orthodox Jewish	26 (18%)	24 (20%)	
Reside in the periphery	44 (29%)	50 (40%)	0.060
Diabetes	3 (2.0%)	4 (3.2%)	0.7
Pre-diabetes	66 (43%)	25 (20%)	< 0.001
Hypertension	5 (3.3%)	0 (0%)	0.066
Dyslipidemia	29 (19%)	15 (12%)	0.10

### Outcomes Data

**Fig. 1:**
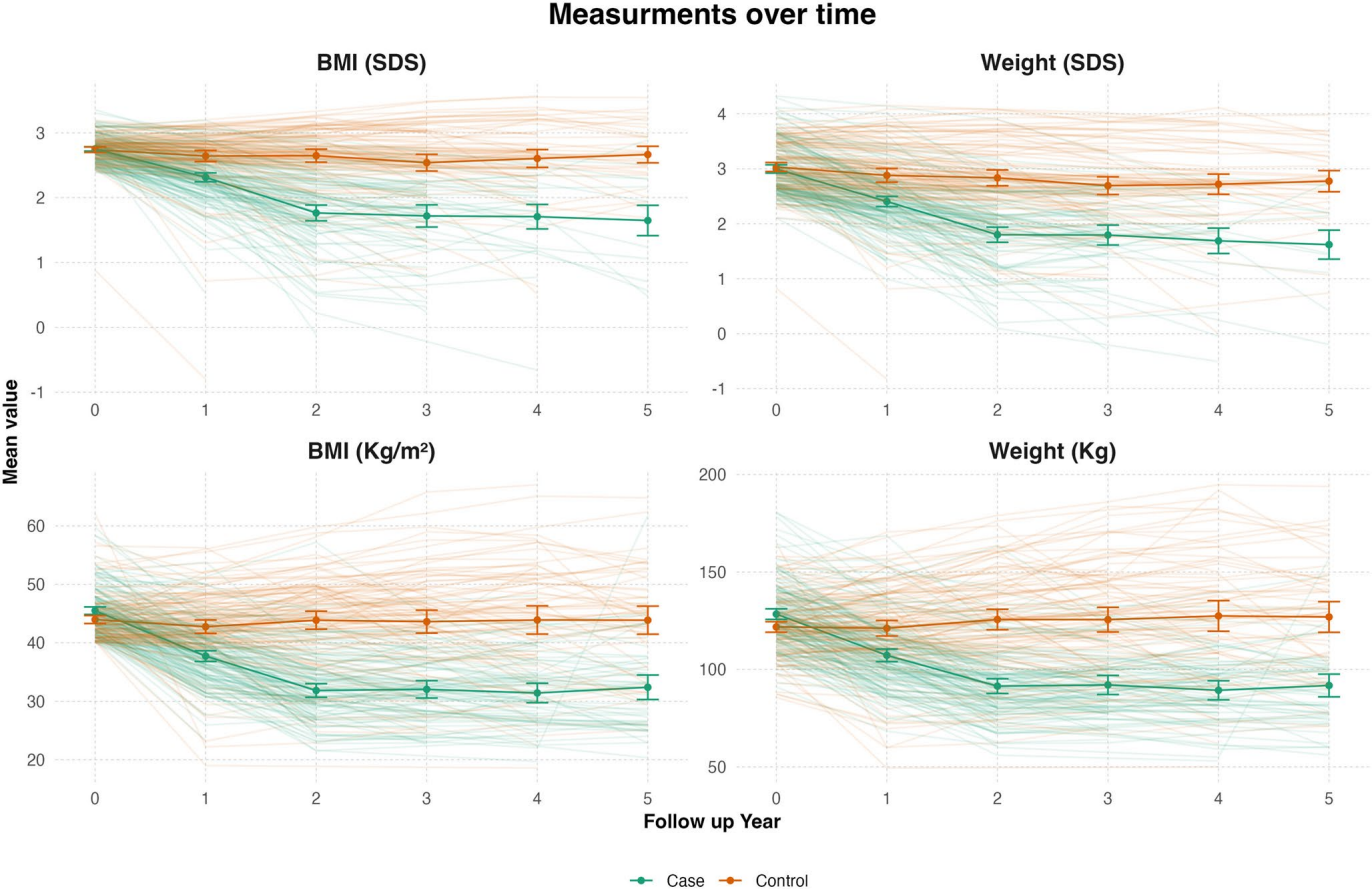
Anthropometric measurements over the 5-year follow-up period

BMI and weight decreased significantly in the surgery group over 5 years (BMI from 44 to 32) compared with the nutritional intervention group (from 45.5 to 44). The nutritional intervention group had a modest decline in BMI in the first year (to 42.7), followed by a plateau. Both BMI and weight SDS significantly drop among the surgery group (BMI: 2.75 to 1.65; Weight: 2.74 to 1.62), while remaining stable in the nutritional intervention group. Complete anthropometric measures are presented in Table 2; Fig. 1, and in supplementary Table 1 S.

**Table 2:** Changes in anthropometric measures over the study period (5 Years)

Characteristic	Missing^1^	Case *N* = 152^1^	Control *N* = 126^1^	*p*-value^2^
BMI				
BMI before index	0 (0)	45.5 (4.0)	44.0 (3.9)	< 0.001
BMI at the first year after index	34 (12)	37.7 (5.3)	42.7 (6.4)	< 0.001
BMI at the second year after index	84 (30)	32 (6)	44 (8)	< 0.001
BMI at the third year after index	144 (52)	32 (5)	44 (9)	< 0.001
BMI at the fourth year after index	160 (58)	31 (6)	44 (10)	< 0.001
BMI at the fifth year after index	173 (62)	32 (8)	44 (9)	< 0.001
Height				
Height before index	0 (0)	168 (8)	166 (9)	0.2
Height at the first year after index	34 (12)	168 (8)	168 (9)	0.6
Height at the second year after index	84 (30)	169 (8)	169 (9)	0.7
Height at the third year after index	144 (52)	169 (9)	170 (10)	> 0.9
Height at the fourth year after index	160 (58)	169 (8)	170 (9)	0.2
Height at the fifth year after index	173 (62)	168 (8)	170 (9)	0.3
Weight				
Weight before index	0 (0)	128 (17)	122 (15)	< 0.001
Weight at the first year after index	34 (12)	107 (19)	121 (22)	< 0.001
Weight at the second year after index	84 (30)	91 (19)	126 (26)	< 0.001
Weight at the third year after index	144 (52)	92 (18)	126 (29)	< 0.001
Weight at the fourth year after index	160 (58)	89 (17)	127 (33)	< 0.001
Weight at the fifth year after index	173 (62)	92 (21)	127 (29)	< 0.001
^1^n (%); Mean (SD)				

^2^Fisher’s exact test; Wilcoxon rank sum test; Wilcoxon rank sum exact test; Welch Two Sample t-test; Pearson’s Chi-squared test

**Fig. 2:**
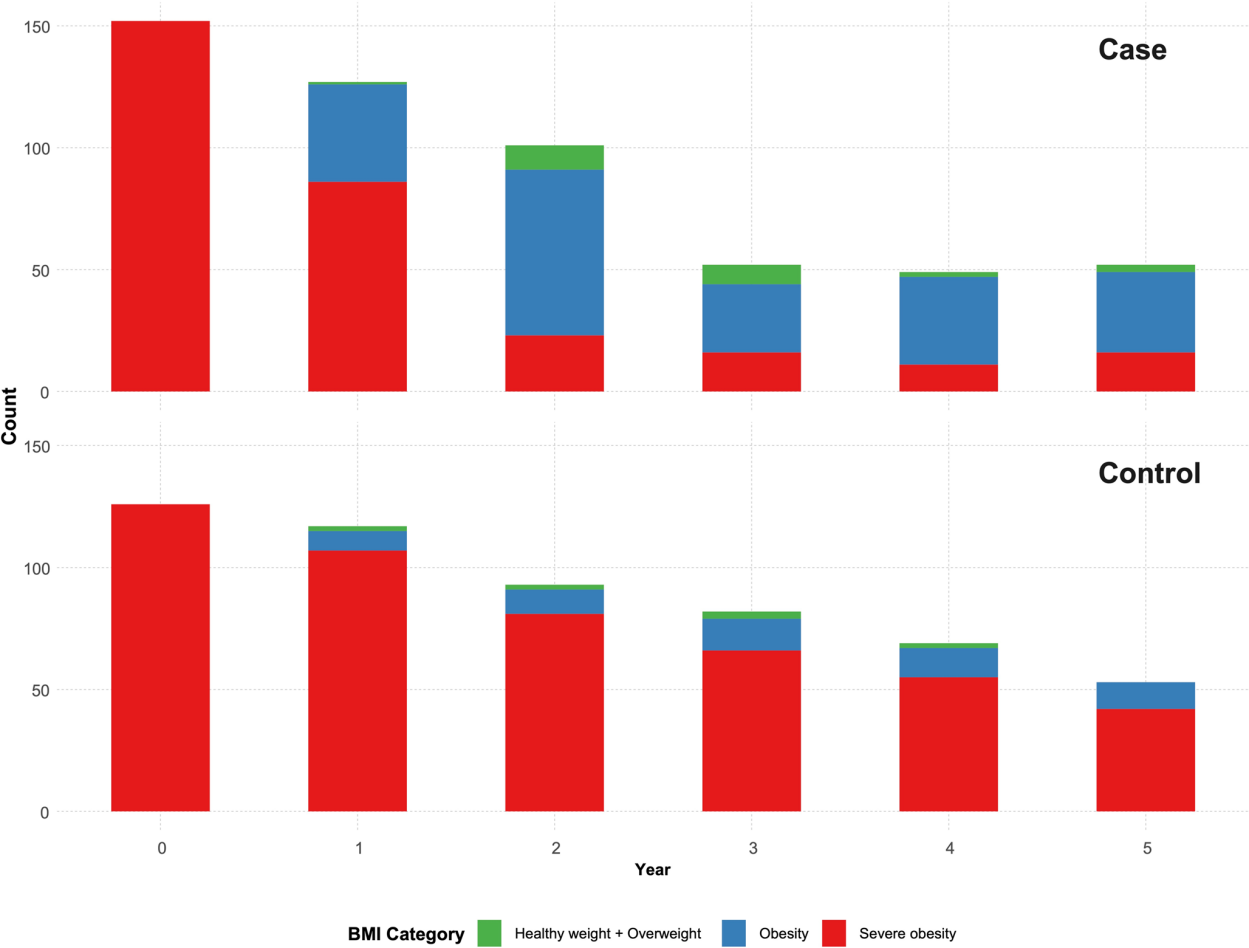
Distribution of Obesity Classes Over Five Years of Follow-Up in the Surgery and Nutritional-Intervention Groups

In the first postoperative year, 31.7% of adolescents in the surgery group—compared with only 8.5% in the nutritional-intervention group—transitioned from severe obesity to a lower obesity class (*p* < 0.001). During years 2–5, approximately three-quarters of those who underwent surgery were in a lower obesity class, whereas only about one-fifth of adolescents in the nutritional-intervention group achieved or maintained such improvement (Fig. 2).

Between-group differences in SDS values were statistically significant after the first year (*p* < 0.001). Multivariate analysis confirmed these trends, showing significant associations between time, group, and outcomes (Table 2S). For weight, both the 0–2 year and 2–5 year periods showed significant declines (*p* < 0.001), with greater reductions in the surgery group (*p* < 0.001). Male participants had higher body weight and BMI. Notably, 13% of the surgery group underwent revision surgery.

Laboratory measurements showed significant differences between groups. Hemoglobin levels were initially similar, but significantly lower among the surgery group from year 2 onwards (year 5: 12.45 vs. 13.46, *p* < 0.001).

**Fig. 3:**
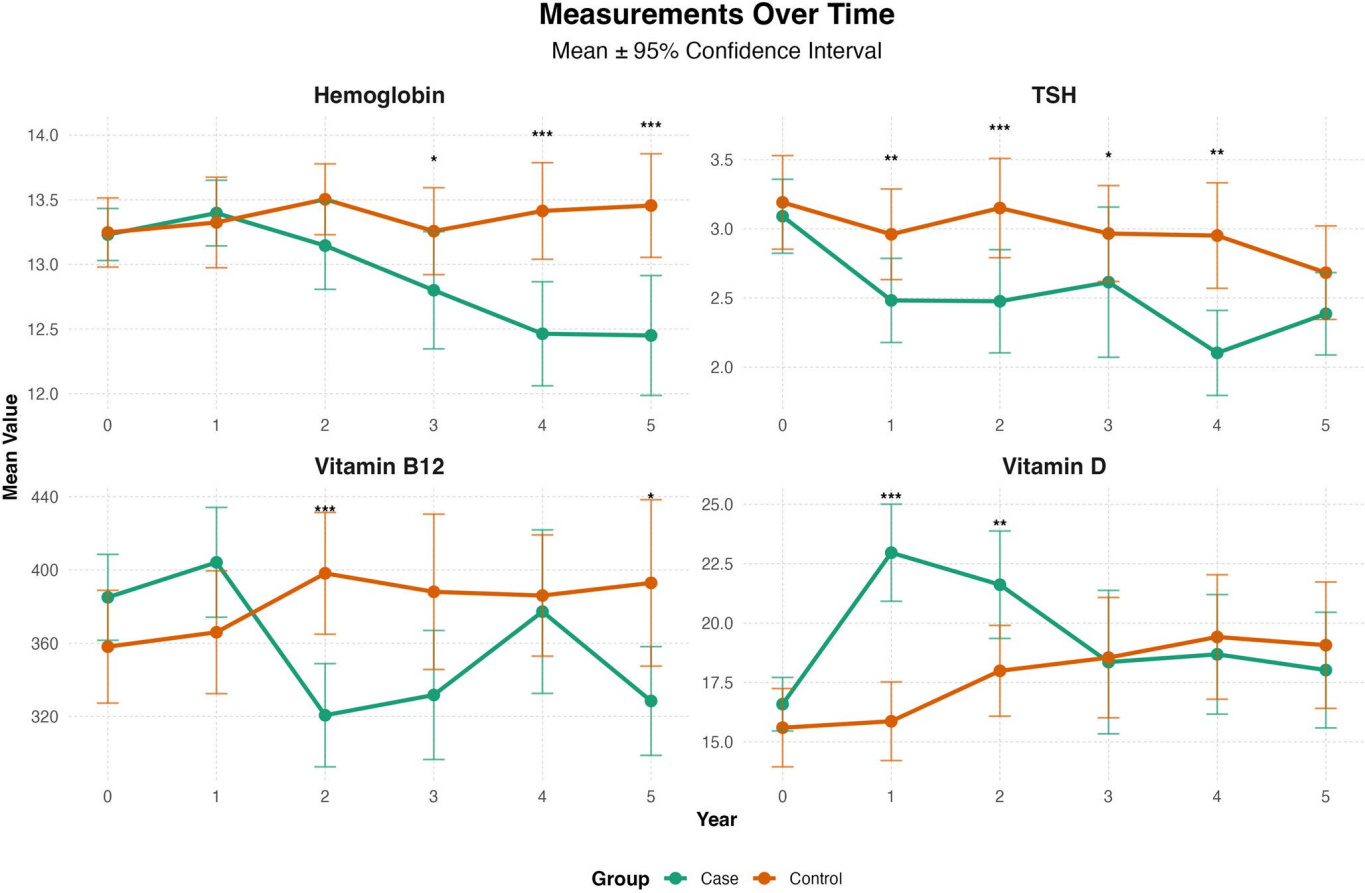
Laboratory measurements over the 5-year follow-up period

Vitamin B12 levels showed a complex pattern. In the univariate analysis, levels were similar between groups, except in the second year, when they were significantly lower in the surgery group (321 vs. 398, *p* < 0.001). In the multivariate analysis, although the overall effects of time and group were not significant, a significant interaction indicated B12 levels declined in the surgery group (*p* < 0.001). Vitamin D levels were initially similar, but higher in the surgery group during the first two years (year 1: 23 vs. 16, *p* < 0.001; year 2: 22 vs. 18, *p* = 0.008). TSH levels declined modestly over time in both groups, with a steeper reduction in the bariatric surgery group. By year 1, mean TSH levels were significantly lower in the surgery group (2.48 vs. 2.96 mIU/L, *p* = 0.006), and this pattern persisted through year 4. In multivariable analysis, there was a significant overall time effect (*p* = 0.03), and a significant year-by-group interaction indicating a steeper decline in the surgery group (*p* = 0.04). Folic acid levels remained similar between groups throughout. The full data is presented in Table 3; Fig. 3, and supplementary Table 2 S.

**Table 3:** Changes in blood tests over the study period (5 Years)

Characteristic	Missing	Case *N* = 152^1^	Control *N* = 126^1^	*p*-value^2^
Hemoglobin Levels				
before index	38 (14)	13.23 (1.25)	13.25 (1.30)	> 0.9
at the first year after index	78 (28)	13.40 (1.39)	13.32 (1.64)	0.9
at the second year after index	113 (41)	13.15 (1.58)	13.50 (1.26)	0.079
at the third year after index	130 (47)	12.80 (1.95)	13.26 (1.51)	0.041
at the fourth year after index	142 (51)	12.46 (1.55)	13.41 (1.69)	< 0.001
at the fifth year after index	156 (56)	12.45 (1.85)	13.46 (1.60)	< 0.001
Vitamin D Levels				
before index	125 (45)	16.6 (5.9)	15.6 (5.8)	0.3
at the first year after index	161 (58)	23 (9)	16 (6)	< 0.001
at the second year after index	179 (64)	22 (8)	18 (7)	0.008
at the third year after index	206 (74)	18 (8)	19 (9)	0.8
at the fourth year after index	203 (73)	19 (7)	19 (9)	> 0.9
at the fifth year after index	195 (70)	18 (8)	19 (9)	0.7
Vitamin B12				
before index	134 (48)	385 (118)	358 (107)	0.2
at the first year after index	128 (46)	404 (151)	366 (123)	0.4
at the second year after index	166 (60)	321 (116)	398 (116)	< 0.001
at the third year after index	185 (67)	332 (123)	388 (147)	0.087
at the fourth year after index	185 (67)	377 (139)	386 (126)	0.5
at the fifth year after index	182 (65)	328 (108)	393 (155)	0.016
TSH Levels				
before index	52 (19)	3.09 (1.61)	3.19 (1.61)	0.8
at the first year after index	115 (41)	2.48 (1.45)	2.96 (1.46)	0.006
at the second year after index	139 (50)	2.48 (1.58)	3.15 (1.53)	< 0.001
at the third year after index	161 (58)	2.61 (1.96)	2.97 (1.45)	0.019
at the fourth year after index	168 (60)	2.10 (1.06)	2.95 (1.56)	0.002
at the fifth year after index	172 (62)	2.39 (1.13)	2.68 (1.23)	0.094
Folic Acid				
before index	146 (53)	5.59 (2.27)	5.35 (2.14)	0.6
at the first year after index	143 (51)	3.60 (2.73)	4.18 (2.73)	0.2
at the second year after index	167 (60)	4.23 (2.58)	4.08 (2.59)	> 0.9
at the third year after index	184 (66)	4.06 (2.61)	4.01 (2.49)	0.7
at the fourth year after index	186 (67)	4.72 (2.48)	3.70 (2.76)	0.078
at the fifth year after index	189 (68)	4.12 (2.35)	3.98 (2.54)	> 0.9
^1^n (%); Mean (SD)				

^2^Fisher’s exact test; Wilcoxon rank sum test; Wilcoxon rank sum exact test; Welch Two Sample t-test; Pearson’s Chi-squared test

## Discussion

### Main Findings

In this study, we report a significant decrease in weight and BMI in adolescents who underwent bariatric surgery, compared with a similar group that received only nutritional intervention, over up to 5 years. We examined trends in vitamin and iron levels, which notably dropped following the surgery.

### Interpretation

We detected a decrease in weight and BMI in the surgery group. The weight decreased from 128 to 92 kg (-28% mean change), whereas in the nutritional intervention group, the weight increased from 122 to 127 kg. Peak weight loss was in the first two years. These observations align with other studies [25 - 27].

The nutritional intervention group remained within the same obesity class throughout the period. Following a weight reduction during the first year (122 to 121 kg), a notable increase was observed in the second year, with average weight rising (126 kg). Nutritional interventions for adolescents with severe obesity are generally considered partially effective [28, 29]. Note that the Center for Disease Control promotes comprehensive family-based programs, emphasizing nutrition, exercise, and behavior. These target children and caregivers and include over 26 h in 2–12 months [30]. This is unlike our intervention. We therefore cannot assume more comprehensive programs are comparable.

Although significant weight and BMI reductions were observed in the surgery group, no meaningful height difference was detected, with no interaction between groups. This suggests surgery does not adversely affect adolescent linear growth, supporting prior evidence that growth potential is preserved [31].

13% of patients in the surgery group underwent revision surgery in this period. All procedures in our study were LSGs, which are associated with lower reoperation rates (around 5%) in adolescents compared to other procedures [25, 32, 33]. This difference warrants investigation into reasons for revision surgeries in our cohort.

Bariatric procedures carry risks of micronutrient deficiencies, so regular monitoring and supplementation are recommended. We observed sustained decline in average hemoglobin levels in the surgery group over the period. Although hemoglobin remained within normal limits, the drop suggests an ongoing nutritional imbalance, likely related to iron deficiency. This may indicate poor supplementation adherence, inadequate intake, or both. While not necessarily symptomatic, the decrease raises concerns about long-term effects. Similar findings have been reported in other studies documenting declines in hemoglobin and iron in adolescents following bariatric surgery [34- 36].

Unlike other micronutrients, vitamin B12 can be stored, and thus, nutritional deficiency may not be apparent for some time. In our study, we observed consistently lower vitamin B12 levels in the surgery group, with a statistically significant difference emerging in the second-year post-surgery; however, values in both groups remained within the normal range. This pattern aligns with previous studies [12], [37 - 39]. This can be attributed to the patients’ reservoir, depleting until it reaches a low in the second year. Another possible explanation is low adherence to supplementation, which is not unprecedented after bariatric surgery [22, 23].

Throughout the period, we observed consistently lower TSH levels in the surgery group, with a statistically significant difference emerging in the second-year post-surgery; however, values in both groups remained within the normal range. This finding aligns with evidence that links obesity to mildly elevated TSH and T3 levels. Following substantial weight loss, like through bariatric surgery, these changes may reverse [40, 41]. Specifically, studies in adolescents have shown that TSH or T3 levels decrease after weight reduction, supporting the hypothesis that obesity-related thyroid hormone alterations are reversible [42].

Both groups in our study had vitamin D deficiency at baseline. In the first two years following surgery, average levels were higher in the surgery group, with a statistically significant difference observed only in the first year; importantly, their values remained within the normal range during this period. From the third year onward, vitamin D levels were similarly low, falling within the deficient range in both the surgery and nutritional-intervention groups. This pattern might be attributed to supplementation, the adherence to which might decrease over time. These findings are notable given the well-documented prevalence of vitamin D deficiency among adolescents undergoing bariatric surgery and the need for long-term supplementation and monitoring [37, 39, 43, 44].

While weight loss is the most “famous” consequence of bariatric surgeries, the nutritional ramifications are long-lasting and can influence patients’ lives and health. It is therefore recommended to commit to long-term supplementation and follow-up protocols. For both, adherence is problematic. The < country name> national registry for bariatric surgeries in adults noted that many do not comply with the required follow-up [45]. Compliance is challenging, especially for adolescents. Regular follow-up with healthcare providers is critical to improving supplement adherence after bariatric surgery [23]. Follow-up visits offer an opportunity to address barriers and to suggest alternatives.

### Strengths and Limitations

This study has several strengths. First, it is a nationwide study that enabled us to examine a significant cohort, representing adolescents who underwent bariatric surgery during the study period. We focused on several markers to comprehensively present post-surgery trends. Second, we compared these individuals to similar counterparts who attempted traditional nutritional interventions and established long-term outcomes for both groups. Third, we described the population undergoing surgery and identified potential pitfalls in their management.

However, this study also has several limitations that should be acknowledged. First, due to small sample sizes, we were unable to match the surgery and nutritional intervention groups. While we took measures to accommodate for this, matching would have been preferable. To account for this, multivariate analyses were performed to control for common confounders. Second, since the average age for bariatric surgery in children in < country name > is 16–17, choosing to use accurate measures for children and excluding measurements once the patients are considered adults was not the obvious choice; yet we believe it produced a better result. Third, five years might not be enough to fully establish the pattern, and future endeavors should consider longer periods. Fourth, we did not assess additional metabolic outcomes such as glucose levels or lipid profiles, which are key indicators of cardiometabolic improvement following bariatric surgery. Fifth, although mental health status is a critical factor influencing treatment adherence and outcomes in adolescents, we did not evaluate or control for its impact in this study. Including these parameters could have provided a more comprehensive evaluation of the surgery’s effectiveness.

## Conclusion

In this study, we compared adolescents with severe obesity who underwent bariatric surgery to a similar group that received nutritional intervention. We compared weight, BMI, and lab results over 5 years after surgery. In the surgery group, we report weight loss and a decrease in BMI in the first two years after surgery, which later plateaus. Additionally, the data show a significant decrease in average hemoglobin levels over the five years, as well as decreases in average B12 and TSH levels compared to the nutritional intervention group. This highlights the importance of supplementation and long-term commitment to management to ensure weight loss and health in young people undergoing bariatric surgery.

## Supplementary Information

Below is the link to the electronic supplementary material.

Supplementary Material 1

Supplementary Material 2

## Data Availability

The datasets generated and analyzed during the current study are not publicly available due to ethical restrictions.
