# Supplementary material for: Effectiveness of Bariatric Surgery Versus Nutritional Interventions in Adolescents: A Retrospective Cohort Study
Source: Obes Surg. 2026 Feb 28;36(4):1523–32. doi: 10.1007/s11695-026-08521-8 (PMC13083463; doi:10.1007/s11695-026-08521-8)
Supplement: Supplementary file 1 — Supplementary Material 1 [file 11695_2026_8521_MOESM1_ESM.docx]

**Table 1S. Changes in Anthropometric Measures Over the study period (5 Years), Standard Deviation Score**

| **Characteristic** | **Missing** | **Case**  N = 152^1^ | **Control**  N = 126^1^ | **p-value**^2^ |
| --- | --- | --- | --- | --- |
| **BMI, Standard Deviation Score** | | | | |
| bmi_sds_0 | 0 (0) | 2.75 (0.21) | 2.74 (0.24) | 0.7 |
| bmi_sds_1 | 34 (12) | 2.31 (0.40) | 2.64 (0.48) | <0.001 |
| bmi_sds_2 | 84 (30) | 1.76 (0.62) | 2.65 (0.49) | <0.001 |
| bmi_sds_3 | 145 (52) | 1.72 (0.63) | 2.54 (0.60) | <0.001 |
| bmi_sds_4 | 190 (68) | 1.71 (0.67) | 2.60 (0.58) | <0.001 |
| bmi_sds_5 | 234 (84) | 1.65 (0.86) | 2.67 (0.47) | <0.001 |
| **Height, Standard Deviation Score** | | | | |
| height_sds_0 | 0 (0) | 0.29 (1.00) | 0.25 (1.05) | >0.9 |
| height_sds_1 | 34 (12) | 0.26 (1.01) | 0.25 (1.09) | 0.9 |
| height_sds_2 | 84 (30) | 0.26 (1.02) | 0.06 (1.07) | 0.3 |
| height_sds_3 | 146 (53) | 0.38 (1.11) | 0.23 (1.14) | 0.5 |
| height_sds_4 | 190 (68) | 0.17 (1.29) | 0.14 (1.09) | 0.7 |
| height_sds_5 | 235 (85) | 0.12 (1.09) | 0.00 (1.11) | >0.9 |
| **Weight, Standard Deviation Score** | | | | |
| weight_sds_0 | 0 (0) | 3.00 (0.47) | 3.03 (0.47) | 0.2 |
| weight_sds_1 | 34 (12) | 2.41 (0.53) | 2.88 (0.69) | <0.001 |
| weight_sds_2 | 84 (30) | 1.80 (0.71) | 2.84 (0.71) | <0.001 |
| weight_sds_3 | 146 (53) | 1.80 (0.67) | 2.69 (0.74) | <0.001 |
| weight_sds_4 | 190 (68) | 1.69 (0.82) | 2.72 (0.78) | <0.001 |
| weight_sds_5 | 235 (85) | 1.62 (0.97) | 2.78 (0.71) | <0.001 |
| ^1^n (%); Mean (SD) | | | | |
| ^2^Fisher's exact test; Wilcoxon rank sum test; Wilcoxon rank sum exact test; Welch Two Sample t-test; Pearson's Chi-squared test | | | | |

**Table 2S. Changes in Anthropometric Measures Over the study period (5 Years), Standard Deviation Score**
