# Supplementary material for: Effectiveness of Bariatric Surgery Versus Nutritional Interventions in Adolescents: A Retrospective Cohort Study
Source: Obes Surg. 2026 Feb 28;36(4):1523–32. doi: 10.1007/s11695-026-08521-8 (PMC13083463; doi:10.1007/s11695-026-08521-8)
Supplement: Supplementary file 2 — Supplementary Material 2 [file 11695_2026_8521_MOESM2_ESM.docx]

**Table 2S. Summary of Linear Mixed Models**

|  | Height | | Weight (sds) years 0-2 | | Weight (sds) years 2-5 | | BMI (sds) years 0-2 | | BMI (sds) years 2-5 | | Hemoglobin | | TSH | | Vitamin B12 | | Vitamin D | |
| --- | --- | --- | --- | --- | --- | --- | --- | --- | --- | --- | --- | --- | --- | --- | --- | --- | --- | --- |
| term | Beta | p value | Beta | p value | Beta | p value | Beta | p value | Beta | p value | Beta | p value | Beta | p value | Beta | p value | Beta | p value |
| Intercept | 1.74 | 0.03 | 4.42 | 0.00 | 4.10 | 0.00 | 3.15 | 0.00 | 3.52 | 0.00 | 10.79 | 0.00 | 4.89 | 0.00 | 463.1 | 0.00 | 2.79 | 0.61 |
| Year | -0.04 | 0.00 | -0.13 | 0.00 | -0.09 | 0.00 | -0.06 | 0.00 | -0.05 | 0.00 | 0.06 | 0.02 | -0.07 | 0.03 | 4.13 | 0.28 | 0.97 | 0.00 |
| Group (Case) | 0.09 | 0.50 | 0.15 | 0.02 | -0.91 | 0.00 | 0.10 | 0.03 | -0.67 | 0.00 | 0.14 | 0.36 | -0.06 | 0.75 | 22.88 | 0.20 | 2.40 | 0.03 |
| Male | -0.27 | 0.03 | 0.55 | 0.00 | 0.22 | 0.02 | 0.29 | 0.00 | 0.23 | 0.00 | 1.79 | 0.00 | 0.21 | 0.18 | -24.84 | 0.06 | -0.26 | 0.76 |
| Age | -0.09 | 0.09 | -0.11 | 0.00 | -0.08 | 0.04 | -0.04 | 0.03 | -0.06 | 0.06 | 0.11 | 0.04 | -0.12 | 0.06 | -5.25 | 0.35 | 0.85 | 0.02 |
| Year: Group | 0.01 | 0.34 | -0.48 | 0.00 | 0.00 | 0.96 | -0.43 | 0.00 | -0.04 | 0.15 | -0.18 | 0.00 | -0.09 | 0.04 | -16.50 | 0.00 | -0.69 | 0.02 |
